# Biotinidase Deficiency: A Reversible Neurometabolic Disorder (An Iranian Pediatric Case Series)

**Published:** 2013

**Authors:** Parvaneh KARIMZADEH, Farzad AHMADABADI, Narjes JAFARI, Sayena JABBEHDARI, Mohammad Reza ALAEE, Mohammad GHOFRANI, Mohammad Mahdi TAGHDIRI, Seyed Hassan TONEKABONI

**Affiliations:** 1 Pediatric Neurology Research Center, Shahid Beheshti University of Medical Sciences, Tehran, Iran; 2 Pediatric Neurology Department, Mofid Children’s Hospital, Shahid Beheshti University of Medical Sciences, Tehran, Iran; 3 Department of Pediatric Endocrinology, Pediatric Neurology Research Center, Shahid Beheshti University of Medical Sciences, Tehran, Iran

**Keywords:** Biotinidase deficiency, Neurometabolic disorder, Developmental delay, Early diagnosis

## Abstract

**Objective:**

Biotinidase deficiency is one of the rare congenital metabolic disorders with autosomal recessive inheritance. If this disorder is diagnosed in newborn period, could be prevented well from mental and physical developmental delay and most of clinical manifestations.

**Materials & Methods:**

The patients were diagnosed as biotinidase deficiency in Neurology Department of Mofid Children’s Hospital in Tehran, Iran, between 2009 and 2012 were included in this study. This study was conducted to define the age, gender, past medical history, developmental status, general appearance, clinical manifestations, neuroimaging findings, and response to treatment in 16 patients with biotinidase deficiency in this department.

**Results:**

In clinical presentation, cutaneous lesions were not found in 37% of the patients and 43% patients had not alopecia. 75% patients had abnormal neuroimaging that in 56% of them, generalized brain atrophy and myelination delay were found. Results of the present study showed the efficacy of biotin in early diagnosed patients with seizure and dermatological manifestations. The seizure and skin manifestations were improved after biotin therapy.

**Conclusion:**

According to the results of this study, we suggest that early assessment and diagnosis have an important role in the prevention of disease progression and clinical signs.

## Introduction

Biotin is a very important vitamin that found in some foods. It plays an important role as cofactor for pyruvate, propionyl-CoA, beta-methylcrotonyl-CoA and two isoenzymes of acetyl-CoA carboxylasein-gluconeogenesis, amino acid catabolism, and fatty acid synthesis (1).

Biotinidase deficiency is a rare and treatable inherited neurometabolic disorder (2) with an estimated incidence of 1:61, 067 population. This disorder in its severe form is much rarer with incidence of 1:137401 (3). Clinical findings of this disorder include neurological (seizure, ataxia, hypotonia, neurodevelopmental delay), dermatological (eczematous skin rash, seborrheic dermatitis), immunological, ophthalmological, respiratory problems (hyperventilation, apnea and laryngeal stridor), and alopecia (1,4). 

Laboratory findings include abnormal organic acids in the urine, metabolic acidosis and elevated lactate and pyruvate levels in blood. Diagnosis can be confirmed by measuring blood biotinidase activity (5,6). Neuroradiological findings include encephalopathy and cerebral atrophy, cerebral edema and bilateral compensatory ventriculomegaly (4). Neurological, cutaneous and neuroimaging finding scan improve or become normal after biotin treatment in biotinidase deficiency (4,7). Some of these symptoms can be

cured but some of the mremain such as hearing loss, ophthalmic defects and mental retardation (8,9).

## Materials &Methods

Patients were diagnosed as biotinidase deficient according to clinical manifestations, developmental milestones, dermatological symptoms, seizures and neuroimaging findings. Diagnosis was confirmed in all the patients based on assessment of biotinidase activity at metabolic disorders reference laboratory in Germany. The results of biotin therapy were assessed in all the patients. The mean dosage of biotin was 5-20 mg/day.

patient’s data were evaluated and categorized as age, gender, development status, general appearance, clinical manifestations, and neuroimaging findings. The data of this observational study were analyzed using descriptive method and no statistical testing was applied.

## Results

Sixteen patients were included in this study. They were 7 males and 9 females and the age range was from 1.5 months to 52 months. all patients were offspring of consanguineous marriage, so that in 13 patients, their parents were first cousin and in 3 other patients, their parents were second cousin. Five patients had a history of neonatal hospitalization because of respiratory distress, icter, seizure, or irritability. 

13 patients had a history of seizure that most common form of seizures was tonic and myoclonic seizures (37.5%). In past medical history, one patient had a history of recurrent vomiting and another one had anorexia; two other patients had a history of recurrent respiratory and urinary infections; one of the patients had a history of loss of consciousness attacks; one had bilateral undescended testicles; and two cases had a history of severe restlessness. In physical examination, 10 patients had cutaneous involvement; 3 had erythematous lesions in pre-orifices (oral and anal), one had cradle cap (Seborrheic dermatitis) lesions, and one had erythematous, maculopapular and crusted lesions. 8 patients had alopecia and one of them had blond hair. 

Weights of 3 patients were less than 5% percentile and 3 other patients had microcephaly (less than 5% percentile). Five patients had motor vision disorders (3 with strabismus and 2 with nystagmus). Hypertonicity was found in 8 patients.

Three patients had abnormal visual evoked potential (VEP) and 4 patients had abnormality in their auditory brainstem response (ABR). In lab data, 8 patients had increased levels of ammonia and lactate. Three cases had high AST and ALT. CBC, VBG (except one patient with acidosis), serum levels of calcium, phosphorus, triglyceride, and cholesterol were normal. Abdominal sonography showed hepatomegaly in one patient.

Electroencephalography (EEG) in 6 patients was abnormal and had not special pattern. In neuroimaging data, 12 patients had abnormal neuroimaging that in 9 patients, generalized brain atrophy and myelination delay were found in brain imaging, CT scan showed multiple calcification in 1 case. One patient had left hemiatrophy, two showed dismyelination in white matter, and one had abnormal signal changes in basal ganglia. 

All of the cutaneous and hair symptoms were cured with biotin therapy after 3 to 6 months. Seizure in all patients was stopped (except one patient that in this patient, seizure was decreased).

In 3 patients with vision and hearing disorders, their symptoms decreased (Table1).

**Table 1:** Patients and Disease Characteristics Before Biotin Therapy in Biotinidase Deficiency Cases

**Patients/Index**	**No.1**	**No.2**	**No.3**	**No.4**	**No.5**	**No.6**	**No.7**	**No.8**	**No.9**	**No.10**	**No.11**	**No.12**	**No.13**	**No.14**	**No.15**	**No.16**
Age (Month)	3	48	8	5	3	3	18	15	3	4	10	14	8	4	1.5	52
Sex	F	F	F	M	M	M	M	F	F	F	M	M	F	M	F	F
Neonatal hospitalization	-	restlessness	-	-	-	restlessness	-	-	Restlessness	-	-	-	-	Seizur, icter	-	Respiratory distress
Development	Delay	Delay	Delay	Delay	Delay	Delay	Delay	Regression	Delay	-	Delay	Regression	Delay	Delay	-	Regression
PMH	Bad odor	Recurrent infection	-	-	-	Anorexia LOC attack	-	Restlessness	Restlessness	Vomiting	Bilateral UDT	-	-	-	-	Recurrent infection
Skin	Maculopapulardiaper rash	Maculopapular	Maculopapular	Maculopapular	Maculopapular	Crusted erythema	-	Maculopapular	Maculopapular	-	-	Skin lesions	-	Erythema	-	-
Hair	Alopecia	-	-	Alopecia	Alopecia	-	-	-	Alopecia	-	Alopecia	Alopecia	Lucid	Alopecia	Alopecia	-
Organomegaly	-	-	-	-	-	-	-	-	-	-	-	-	-	-	-	-
Consanguineous Marriage	First cousin	First cousin	First cousin	First cousin	First cousin	First cousin	First cousin	econd cousin	First cousin	First cousin	econd cousin	First cousin	First cousin	First cousin	second cousin	First cousin
Weight	Nl	Nl	Nl	Nl	Nl	Nl	dec.	Nl	Nl	dec.	dec.	Nl	Nl	Nl	Nl	Nl
HC	Nl	dec.	Nl	Nl	Nl	Nl	Nl	Nl	dec.	Nl	Nl	Nl	Nl	dec.	Nl	Nl
Eye movement	Nl	Strabism	Nl	Nystagmus	Nl	Nl	Nystagmus	Nl	Nl	Nl	Nl	Nl	Nl	Nystagmus	Nl	Strabism
Visuality	dec	Nl	Nl	Nl	Nl	Nl	Nl	Nl	Nl	Nl	Nl	Nl	Nl	Nl	Nl	dec.
Movement Disorder	_	-	-	-	-	Myoclonus	-	Dystonia	-	-	-	-	-	-	-	-
Tonicity	dec	inc.	dec.	inc.	inc.	inc.	inc.	inc.	-	dec.	inc.	inc.	-	-	-	dec.
DTR	Nl	Nl	Nl	inc.	inc.	inc.	inc.	inc.	Nl	Nl	inc.	Nl	Nl	Nl	Nl	dec.
Seizure	Infantile spasm	-	+	+	+	Myoclonic	Resistant partial	-	+	Tonic-myoclonic	Tonic	Myoclonic	-	+	Tonic	+
Lactate	Nl	inc.	Nl	inc.	inc.	Nl	inc.	Nl	inc.	inc.	Nl	Nl	inc.	Nl	Nl	inc.
Ammonia	Nl	Nl	Nl	Nl	Nl	Nl	Nl	Nl	Nl	Nl	Nl	Nl	Nl	Nl	Nl	Nl
Pyruvate	Nl	Nl	Nl	Nl	Nl	Nl	Nl	Nl	Nl	Nl	Nl	Nl	Nl	Nl		Nl
ALT	Nl	inc.	Nl	Nl	Nl	inc.	Nl	Nl	Nl	Nl	Nl	Nl	Nl	Nl	inc.	Nl
AST	Nl	inc.	Nl	Nl	Nl	inc.	Nl	Nl	Nl	Nl	Nl	Nl	Nl	Nl	inc.	Nl
ABR	Disturbed	Disturbed						Nl				Nl	Nl	Severely disturbed	Nl	Disturbed
EEG	Moderate	Mild	Nl	Nl	Nl	Nl	Nl	Nl	Moderate	-	Mild	Nl	Nl	Nl	Mild	Mild
Imaging changes	yelination delay	trophy	-	trophy	-	-	Hyper-intensity in white matter on T2 MRI	-	yelination delay	Myelinationn delay	ultiple calcification in CT, normal MRI	evere brain atrophy	trophy	rain atrophy	Left hemi-atrophy, generalized atrophy	Brain atrophy, subdural effusion
VEP		isturbed		Nl	Nl		Nl		isturbed	Nl		Nl	Nl	Disturbed	Nl	

Abbreviations: F, female; M, male; Nl, normal; dec, decrease; inc, increase; PMH, past medical history; HC, head circumference; DTR, deep tendon reflex; VEP, visual evoked potential; EEG, electroencephalography; Mild, mildly abnormal (involvement<30%); Moderate, moderately abnormal (involvement>30% but not all of the EEG trace)

**Fig 1:**
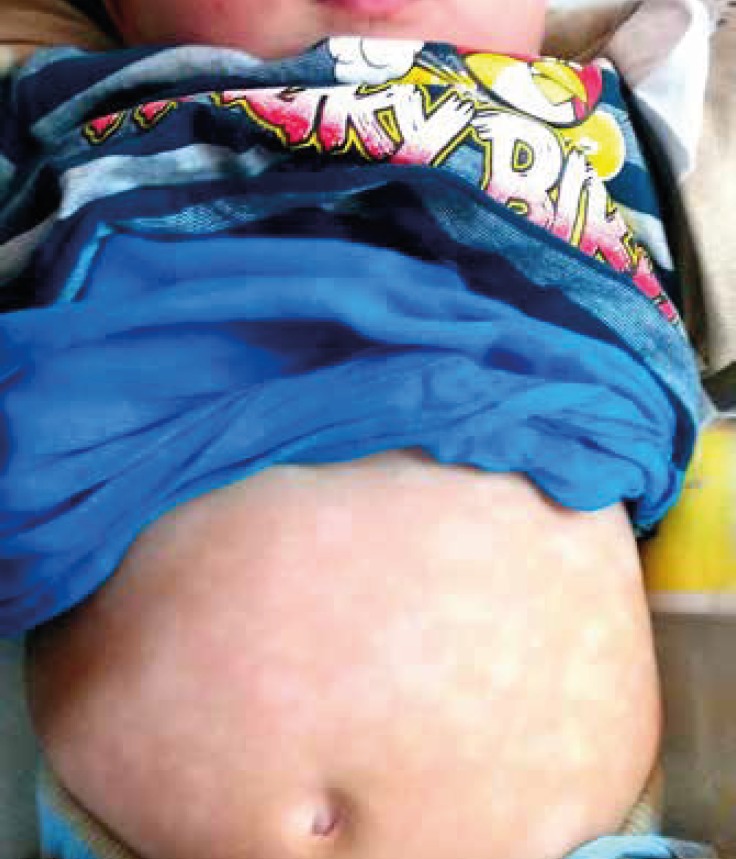
4 month -Boy- case of biothinidase deficiency pre treatment

**Fig2:**
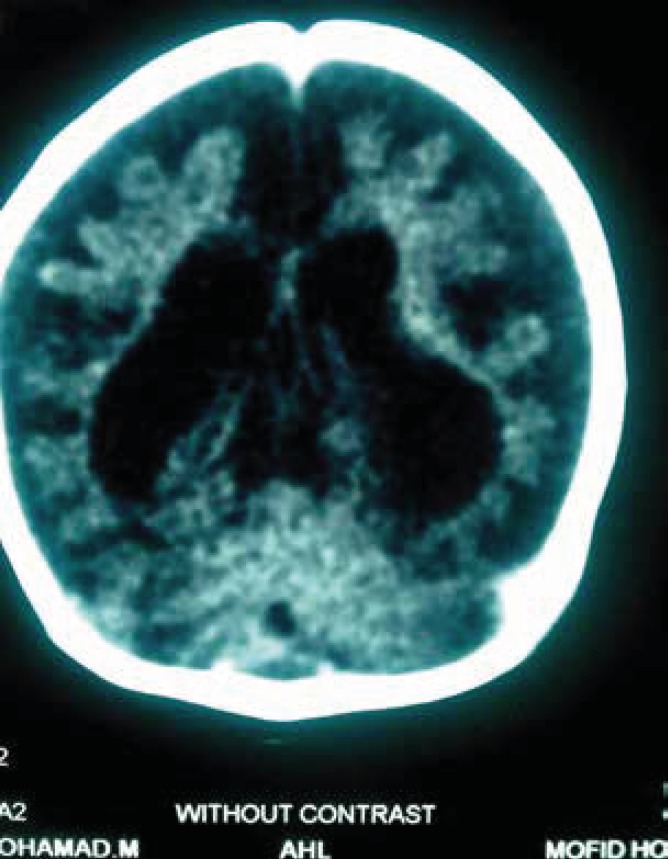
Case of biothinidase deficiency-with severe brain atrophy

**Fig3:**
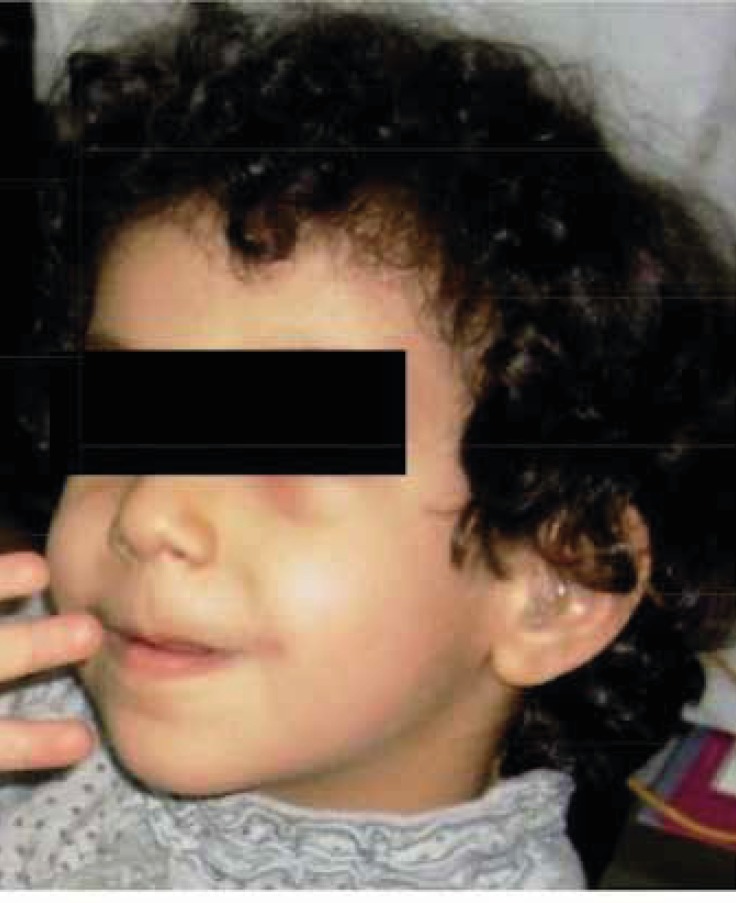
50 month-Girl-case of biothinidase deficiency after treatment

## Discussion

The results of this study demonstrated that biotin therapy in patients with biotinidase deficiency can reduce, prevent or improve neurological, dermatological and other manifestations of biotinidase deficiency. Dermatological manifestations included alopecia, loss of hair color, hypopigmentation, and eczematous and erythematous perioral and perianal papules. These findings are secondary to abnormal fatty acid synthesis because of carboxylase deficiency. Dermatological manifestations responded to administration of biotin 3 to 6 months after initiation of the treatment. The most frequent seizure types in patients with biotinidase deficiency were tonic and myoclonic. Generalized tonic seizures were seen in three patients and myoclonic seizures were seen in three cases and all of them were resolved with biotin therapy. It is important that seizures did not respond to conventional therapies, but had rapid improvement in response to biotin therapy. Cutaneous symptoms and neurodevelopmental delay were also improved after treatment with biotin.

These findings are similar to the results of studies by Wolf and Grunewald et al. that reported biotin therapy in biotinidase deficient infants with seizures (untreatable form of seizures with antiepileptic drugs), cutaneous manifestation, visual and auditory abnormalities, neuroradiological findings such as encephalopathy, and lab data such as elevated blood lactate and pyruvate concentrations is important to reduce these manifestations (1,10). If biotinidase deficient patients do not treated by biotin at early stages or infancy, irreversible neurological damage, dermatological manifestations and other symptoms will progress (11).

Symptoms of biotinidase deficiency in this study are similar to previous study that contained cutaneous lesions, alopecia, neurodevelopmental delay, and brain atrophy, but in our study, there were special notices, such as all patients were the offspring of consanguineous marriage. Cutaneous lesions were not found in 6 patients and 7 patients did not have alopecia and symptoms in their hair.

MRI in half of the patients was abnormal. Cutaneous symptoms were cured with biotin therapy, and neurological symptoms such as seizure, vision and hearing impairments improved.

**In conclusion, **this study demonstrated that using biotin as an early treatment in biotinidase deficiency has a therapeutic effect in patients with this reversible neurometabolic disorder. Our study showed that all patients were the offspring of consanguineous marriage.

Cutaneous lesions were not found in 37% of patients and 43% patients did not have alopecia or any other symptoms in their hair, so absence of these symptoms do not reject the existence of biotinidase deficiency.
